# The complete chloroplast genome sequence of *Heracleum millefolium* Diels (Apiaceae)

**DOI:** 10.1080/23802359.2022.2086073

**Published:** 2022-06-30

**Authors:** Dunzhu Ciren, Chen Fan, Xiang Liu, Zhiwei Zhang, Jiashui Wang, Lan Cao

**Affiliations:** a University of Tibetan Medicine, Lhasa, China; b School of Medicine and Chemical Engineering, Yangling Vocational & Technical College, Xianyang, Shsaanxi, China; c Chongqing Academy of Chinese Materia Medica, Chongqing, China; d Research Center for Traditional Chinese Medicine Resources and Ethnic Minority Medicine, Jiangxi university of Chinese Medicine, Nanchang, China

**Keywords:** Plastome, medicinal plant, phylogeny; *Heracleum*

## Abstract

We assembled the complete chloroplast genome of *Heracleum millefolium* which is a traditional widely used medicinal plant in China. The whole genome is 150,025 bp in length which was divided into four subregions: a large single-copy region (93,645 bp), a pair of 19,458 bp inverted repeats regions, and a small single-copy region (17,464 bp), respectively. Additionally, the chloroplast genome of *H. millefolium* detected 128 genes, including 85 protein coding genes, 36 transfer RNAs, and eight ribosomal RNAs. The overall GC content of this chloroplast genome is 37.5% and the mean coverage value is 1752.4x. Phylogenetic analysis based on 17 chloroplast genomes dataset was conducted to clarify the relationships of the major clades in Apiaceae. The results strongly supported the monophyly of *Heracleum* and the closer relationship of *H. millefolium* and *H. candicans*.

*Heracleum millefolium* Diels 1906 is a perennial herb mainly distributed in Southwestern China and occur in sparse forests, forest margins, alpine scrub and meadows at an altitude of 2,800–5,000 m which subjects to the genus *Heracleum* L., Tordylinae in Apiaceae family (She et al. 2005)*. Heracleum millefolium* is used as *H. hemsleyanum*, which is a traditional widely used medicinal plant and its root is used to treat numbness in waist and knees, limb cramps and vitiligo (Wu 1988), and it was ranked as ‘Least Concern’ (LC) in the latest Chinese Higher Plants Red List (Qin et al. 2017). The genus *Heracleum* has about 70 species and is a widespread, taxonomically complex genus with the Hengduan Mountains forming one of two centers of diversity (She et al. 2005) while there has limited molecular data in the GenBank for the taxonomy research on this genus. Complete chloroplast (cp) genome sequences could provide abundant informative molecular evidence to resolve the intractable taxonomic issues (Firetti et al. 2017; Niu et al. 2018). Therefore, we here reported the cp genome sequence of *H. millefolium* to provide molecular data for the researches focusing on the classification or conservation of the *Heracleum* species.

We sampled mature leaves of *H. millefolium* from Changdu, Xizang Autonomous Region, China (30.131 N, 98.069E, altitude 4,310 m) and fresh leaves were quickly dried with silica gel for DNA extraction. The voucher specimen is deposited at the Herbarium of Chongqing Academy of Chinese Materia Medica (Lan Cao; caolanf625@163.com) under the Voucher number 542126LY0348. Materials was then sent to Novogene (Beijing) for DNA extraction, library construction and sequencing. Paired-end reads of 2 × 150 bp for the sample were generated in a single lane on an Illumina HiSeq2500 sequencer. The raw data (6 G) obtained from Novogene were filtered using Trimmomatic v0.3.2 with default settings (Bolger et al. 2014). The clean reads were assembled using the program NOVOPlasty (Dierckxsens et al. 2017) with the chloroplast (cp) genome of *H. yungningense* as the reference (MN893285; Zheng et al. 2020). The reconstructed cp genome was annotated using the Geneious annotation tool with the cp genome of *H. yungningense* as the reference. Finally, we used OGDRAW (Lohse et al. 2013) to draw circular cp genome map. The annotated cp genome sequence has been submitted to the GenBank (accession number: MW228410).

The complete chloroplast genome of *H. millefolium* was 150,025 bp in length with a mean coverage value of 1752.4x. The GC contents was 37.5%. Four distinct sub-regions were separated within the complete chloroplast: the large single copy (LSC) region (93,645 bp), small single copy (SSC) region (17,464 bp), and a pair of inverted repeat regions (19,458 bp). The chloroplast genome contains a total of 128 genes including 85 protein coding genes, 36 tRNA genes, and eight rRNA genes. Additionally, we generated a maximum-likelihood tree (ML) based on cp genomes of 17 species (12 genus from Apiaceae: *Angelica*, *Apium*, *Bupleurum*, *Cicuta*, *Heracleum*, *Ligusticum*, *Ostericum*, *Peucedanum*, *Pimpinella*, *Prangos*, *Saposhnikovia* and *Seseli*) (Figure 1) to clarify the phylogenetic relationships between *H. millefolium* and other species in Apiaceae using RaxML (Stamatakis 2006) with 1,000 bootstrap replicates. The complete cp genome sequences of the17 species including the outgroup *Panax notoginseng* (MK408955) was aligned using MAFFT (Katoh and Standley 2013). The phylogenetic analysis results strongly supported the monophyly of *Heracleum* and the closer relationship of *H. millefolium* and *H. candicans* (Figure 1).

**Figure 1.**
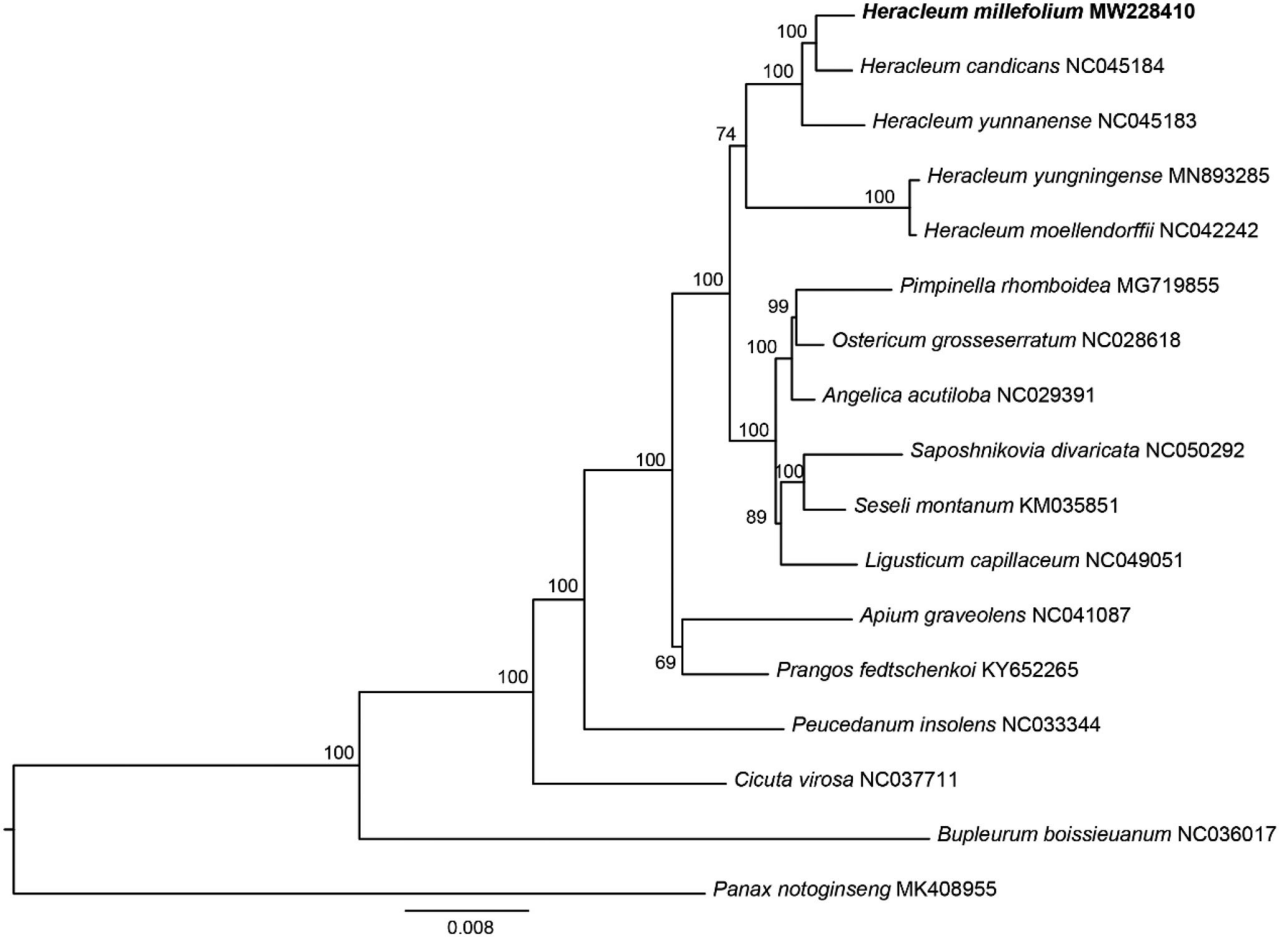
Phylogenetic relationships in Apiaceae based on chloroplast genome data from 17 species with *Panax notoginseng* was selected as outgroup. Numbers on the nodes are bootstrap values from 1,000 replicates.

## Data Availability

The genome sequence data that support the findings of this study are openly available in GenBank (https://www.ncbi.nlm.nih.gov) under the accession no. MW228410. The associated BioProject, SRA, and Bio-Sample numbers are PRJNA789940, SRR17267053, and SAMN24175437, respectively.

## Sampling statement

Our materials are not listed in the Wild Plants Under State Protection in China, so we need not any permission or license to collect them; and we only sampled a few leaf materials for molecular experiment and didn't damage any plant.
